# The SIMULATE ureteroscopy training curriculum: educational value and transfer of skills

**DOI:** 10.1007/s00345-021-03604-w

**Published:** 2021-02-03

**Authors:** Abdullatif Aydın, Kamran Ahmed, Umair Baig, Nicholas Raison, Andrea G. Lantz Powers, Nicola Macchione, Ahmed Al-Jabir, Takashige Abe, Muhammad Shamim Khan, Prokar Dasgupta, the SIMULATE Trial Contributors

**Affiliations:** 1 MRC Centre for Transplantation, King’s College London, King’s Health Partners, London, UK; 2 Department of Urology, King’s College Hospital NHS Foundation Trust, King’s Health Partners, London, UK; 3 Department of Urology, Dalhousie University, Halifax, NS Canada; 4 Department of Urology, ASST Santi Paolo E Carlo, Università Degli Studi Di Milano, Milan, Italy; 5 Department of Urology, Hokkaido University Graduate School of Medicine, Sapporo, Japan; 6 Urology Centre, Guy’s and St. Thomas’ NHS Foundation Trust, King’s Health Partners, London, UK

**Keywords:** Education, Simulation, Urology training, Ureterorenoscopy, Curriculum

## Abstract

**Objective:**

Different simulation modalities may be utilised in a curricular fashion to benefit from the strengths of each training model. The aim of this study is to evaluate a novel multi-modality ureterorenoscopy (URS) simulation curriculum in terms of educational value, content validity, transfer of skills and inter-rater reliability.

**Methods:**

This international prospective study recruited urology residents (*n* = 46) with ≤ 10 URS experience and no prior simulation training. Participants were guided through each phase of the expert-developed SIMULATE URS curriculum by trainers and followed-up in the operating room (OR). Video recordings were obtained during training. A post-training evaluation survey was distributed to evaluate content validity and educational value, using descriptive statistics. Performance was evaluated using the objective structured assessment of technical skills (OSATS) scale to measure improvement in scores throughout the curriculum. Pearson’s correlation coefficient and Cohen’s kappa tests were utilised to investigate correlation and agreement between raters.

**Results:**

Participants reported gaining OR-transferrable skills (Mean: 4.33 ± 0.67) and demonstrated marked improvement in throughout the curriculum, transferred to the OR for both semi-rigid URS (*p* = 0.004) and flexible URS (*p* = 0.007). 70% of participants were successfully followed-up in the OR (*n* = 32). No differences were identified with the additional use of fresh frozen cadavers (*p* = 0.85, *p* = 0.90) and the URO Mentor VR simulator (*p* = 0.13, *p* = 0.22). A moderate level of correlation was noted on the video OSATS assessments, between two expert assessors (*r* = 0.70), but a poor agreement with the live rating.

**Conclusion:**

The SIMULATE URS training curriculum received high educational value from participants, who demonstrated statistically significant improvement with consecutive cases throughout the curriculum and transferability of skills to the OR in both semi-rigid and flexible URS.

**Supplementary Information:**

The online version contains supplementary material available at 10.1007/s00345-021-03604-w.

## Introduction

Surgical simulation is believed to be an effective adjunct to enhance performance in the operating room (OR) [1–4] during the initial phase of training. Since its early days, several modalities have been utilised in generic and procedural skills training [1] including synthetic dry-lab (bench) models, animal models, human cadavers and virtual reality (VR) simulators. Each type of modality or model presents different strengths and weaknesses [5]. Thus, it is imperative to identify these for models described and/or validated in the literature, for the suitability of training, and subsequently, develop structured multi-modality simulation-based curricula. Ureterorenoscopy (URS) is a prime example of a core procedure with multiple different training models described in the literature [6–8].

SIMULATE (Simulation in Urological Training and Education) is the first multi-institutional superiority randomised controlled trial (RCT) evaluating whether surgical trainees who undergo additional simulation training, compared to standard OR-based training, are able to achieve proficiency sooner and with improved patient outcomes [9]. As part of this study, we developed a comprehensive simulation-based training curriculum for novice trainees with minimal URS experience. Herein, we report the evaluation of the SIMULATE URS training curriculum in terms of educational value, content validity, transfer of skills and inter-rater reliability.

## Methods

Ethical approval was obtained, as part of the SIMULATE project (BDM/14/15–68) [9].

### Study process

**Fig. 1:**
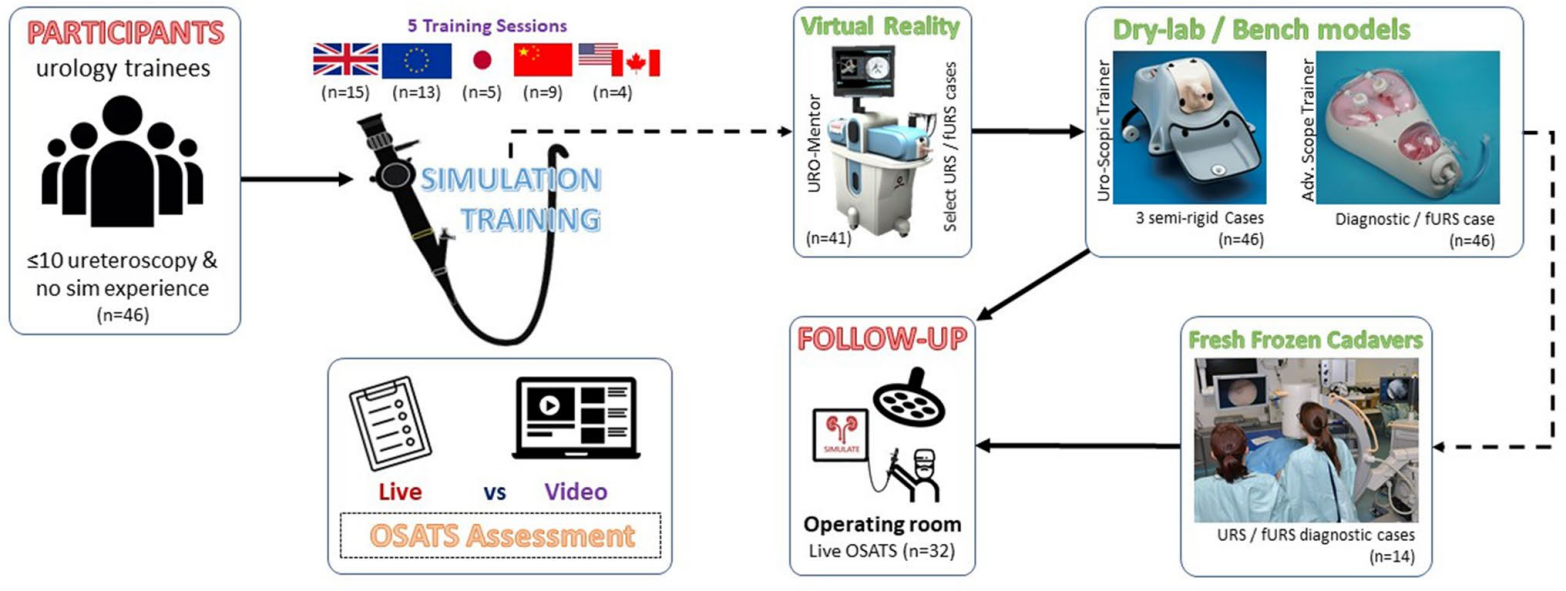
An overview of the study methodology, the utilised training curriculum and participant timeline. Five training sessions were conducted with novices in ureteroscopy (*n* = 46). Performance evaluation took place live and through video assessments. Participants were followed-up in their home institutions for transfer of skills. *OR* operating room, *OSATS* objective structured assessment of technical skills, *URS* ureteroscopy, *fURS* flexible ureterorenoscopy

Newly appointed residents with experience of 0–10 procedures and no structured simulation training in URS were recruited from UK deaneries as well as centres from Austria, Germany, Greece, Switzerland, Turkey, China, Japan, Canada and the United States of America. Training sessions were conducted for the simulation arm of the RCT [9] utilising an expert-developed training curriculum on five separate occasions (Fig. 1). Participants were guided through each phase of the curriculum with local board-certified urologists. Live OR assessment was requested as follow-up from each participants’ supervisor, who were blinded to whether they received simulation training, as part of the greater trial [9].

### Training curriculum

Available training models with the highest levels of evidence for semi-rigid and flexible URS were identified as URO-Mentor (Simbionix, Israel) VR simulator, Uro-Scopic Trainer (Limbs and Things, UK) and Scope Trainer (Mediskills, UK) [10]. A Delphi process was undertaken for curriculum development, with input from experts and trainees in urolithiasis, as previously described [9].

The curriculum begins with didactic lectures including:

- Set up, ureteric access and retrograde studies
- Guidewires, access sheaths, stents and baskets
- Lasers—types, size, settings and safety
- Semi-rigid ureteroscopy—indications, technique and risks/complications
- Flexible ureterorenoscopy—indications, technique and risks/complications

It continues with select tasks and cases on the URO-Mentor VR simulator, suitable cases on bench models and diagnostic cases (Fig. 1) on fresh frozen cadavers (FFC), if locally available. All efforts were made to keep training standardised across sites, in line with the developed curriculum. Didactic lectures and supervision took place in multiple languages, according to the site of delivery. FFCs could only be utilised for UK participants (*n* = 14) and VR Simulation was unavailable for Japanese trainees (*n* = 5); however, participants were taught these tasks and cases on dry-lab models to compensate.

### Performance evaluation

Throughout training sessions, supervising faculty assessed participants on their performance using the well-validated objective structured assessment of technical skills (OSATS) assessment scale [11]. Video recordings of performances were also obtained and distributed to two urolithiasis experts for the blind assessment using OSATS. All trainees were invited to complete an anonymous post-training evaluation survey, asking to rate different aspects of training on a 5-point Likert scale.

### Outcome measures

The primary outcome of this study is to report the educational value of the SIMULATE URS curriculum. Other outcome measures were content validity, transfer of skills and inter-rater reliability. Educational value was measured using participant opinion through post-training surveys. Acquisition and transfer of skills were measured by improvement in candidate performance through the curriculum and the OR, as per OSATS scores. Measurement of inter-rater reliability was conducted between the two experts as well as live rating by faculty. Secondary analyses were performed to compare the OR performances of those exposed to FFC (*n* = 14) vs non-FFC (*n* = 32) and VR (*n* = 41) vs non-VR (*n* = 5) groups to further evaluate these expensive components of the curriculum.

### Statistical analysis

Descriptive statistics were used for questionnaire data. OSATS scores were converted to percentages since there were aspects which could not be rated through video assessments such as “Use of Assistants” and Knowledge of Instruments”. Pearson’s correlation coefficient and Cohen’s kappa tests were utilised to investigate correlation and agreement, respectively, in scores for inter-rater reliability, using SPSS^®^ Statistics version 26 (IBM^®^, Armonk, NY, USA). A mean OSATS score was used for the remainder analyses. GraphPad Prism version 8 (San Diego, CA, USA) was utilised for all graphs and perform other basic statistical tests. Following the establishment of normality through Shapiro–Wilk tests, unpaired *t *tests were performed to compare differences in performance between participants who were exposed to FFC, not exposed to VR and those that only trained using VR and bench models. A one-way ANOVA test was used to measure improvement in OSATS scores over the curriculum. A *p *value < 0.05 was considered statistically significant for all tests.

## Results

### Demographics

This study recruited a total of 46 participants (Fig. 1), supervised by a total of 19 specialists over the five conducted sessions. Participants were all residents, in years 1–4, enrolled onto a urology training programme, with ages ranging between 24–43 years (Mean 29.4 ± 3.7). 11 of 46 participants were female (24%). As per our eligibility criteria, all recruits were novices in URS and had performed a mean of 2 semi-rigid (Range 0–9) and 1 flexible URS (Range 0–7) procedures. 70% of participants returned follow-up data in the OR (*n* = 32), as assessed by their local supervisors, using OSATS.

### Educational value

The training programme was evaluated by participants on a Likert scale (1 = ‘Strongly Disagree’, 5 = ‘Strongly Agree’) and well received. Respondents stated that their skills improved upon completing the training curriculum (Mean 4.39 ± 0.78) and that they gained transferrable skills for the OR (Mean 4.33 ± 0.67). Furthermore, trainees thought that simulation-based training is essential for patient safety (Mean 4.56 ± 0.62) and that there is a role for further dedicated procedural training curricula (Mean 4.56 ± 0.62).

### OSATS performance

**Fig. 2:**
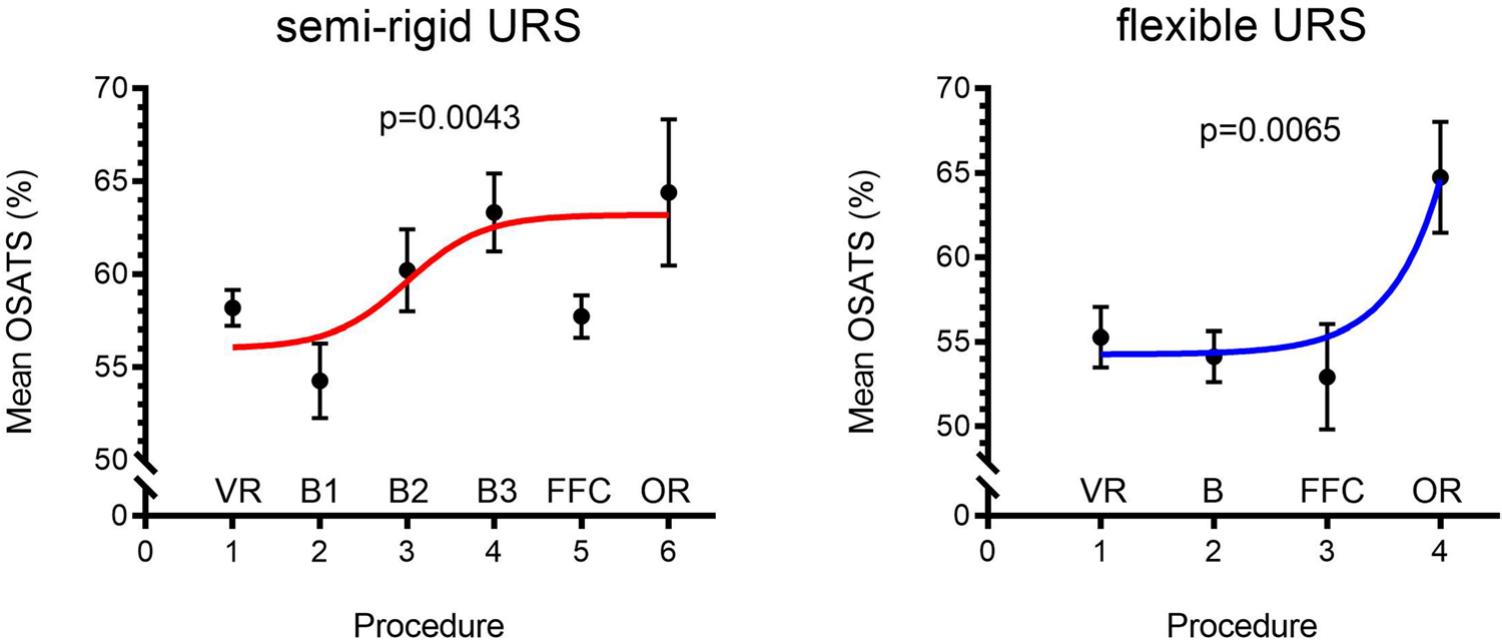
Improvement in mean OSATS (%) scores over consecutive scenarios and operating room (OR). *B* Bench, *FFC* fresh frozen cadavers, *OR* operating room, *VR* virtual reality

There was marked improvement in performance throughout the curriculum (Fig. 2) and transferred to the OR for both semi-rigid URS (*p* = 0.0006) and flexible URS (*p* = 0.0003). Technical performance was observed to be particularly low when participants performed cases on FFCs for both semi-rigid (Mean 58% ± 4) and flexible URS (Mean 53% ± 12) alike. In terms of semi-rigid URS, no differences were observed in OR performance between trainees exposed to FFCs (*n* = 9, Mean: 70.1% ± 14) and those who trained using VR and bench models (*n* = 18, Mean 70.6%; *p* = 0.95). Similarly, no statistically significant differences were observed between those not exposed to VR (*n* = 5, Mean 52.0%) and trainees who trained on VR and Bench (*n* = 18, Mean 70.6%; *p* = 0.07). Likewise for flexible URS, both comparisons were insignificant for FFC vs non-FFC [(*n* = 9 vs *n* = 16), (Mean 67.7% vs 69.9%), *p* = 0.79] and non-VR vs VR and bench [(*n* = 5 vs *n* = 16), (Mean 52.6% vs 69.9%); *p* = 0.10].

### Inter-rater reliability

**Fig. 3:**
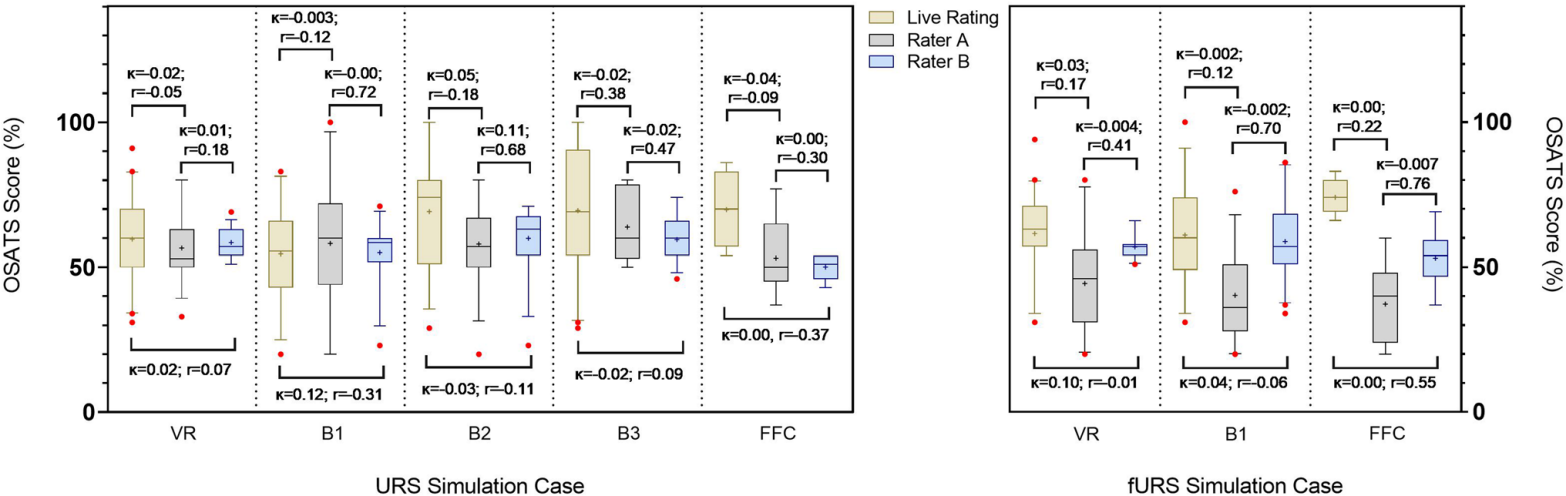
Inter-rater reliability of all ureteroscopy cases between the video assessment of two expert raters (**a**, **b**) and live rating. A Pearson’s coefficient and Cohen’s kappa was performed for all cases to assess correlation and agreement, respectively. *B* bench, *FFC* fresh frozen cadavers, *OR* operating room, *VR* virtual reality

Comparisons were made between live OSATS rating and the two experts video assessors (Fig. 3). There was generally a poor agreement between all three parties, but moderate correlation between the video assessments of experts A and B. Strong correlation was observed between raters A and B for semi-rigid URS Bench-1 (*r* = 0.72) and Bench-2 (*r* = 0.68) cases as well as fURS Bench (*r* = 0.70) and Cadaveric (*r* = 0.76) cases. There was one isolated case of fair correlation between the live raters and rater B (*r* = 0.55) for fURS cadaveric case.

### Content validity

Using a Likert score (1 = ‘Least Useful’, 5 = ‘Most Useful’), participants rated various aspects of the utilised simulators ≥ 3/5 for suitability (Supplementary Figure). The most highly rated modality was FFCs (Mean 4.10 ± 0.55), of which the highest-rated aspect was C-arm control (Mean 4.72 ± 0.46). Stent insertion was rated relatively lower (Mean 3.06 ± 1.39) and further explanations noting this task proved to be difficult due to the ureters being frozen was provided as qualitative feedback. This was followed by Uro-Scopic Trainer (Mean 3.88 ± 0.31), Advanced Scope Trainer (Mean 3.59 ± 0.33) and the URO-Mentor (Mean 3.57 ± 0.28). Both the dry-lab bench models scored highly in instrument handling, stone fragmentation and stone extraction. The URO-Mentor VR simulator scored a mean of 3.9 on ureteric navigation, stone extraction and stent insertion. Qualitatively, it was reported that flexible URS tasks were particularly useful on this simulator.

## Discussion

Recent developments in surgical education suggest that models should be utilised in combined curricula which address specific learning needs [12]. As such, a simulation curriculum was developed for the SIMULATE RCT [9], utilising input from urolithiasis experts and trainees to be delivered as an intervention for the simulation cohort. This study reports the validity assessment of the developed curriculum and transfer of technical skills to the OR.

Participants who trained through our curriculum demonstrated good progress in OSATS performance and transfer of skills to the OR in both semi-rigid and flexible URS (Fig. 2). Trainee perception, from the educational value survey, also correlate well with the skills performance. A similar modular curriculum was described by Soria et al. [13, 14] for semi-rigid [13] and flexible URS [14], which also begins with theoretical knowledge followed by simulation using the ETXY Uro-Adam (ProDelphus) bench model and a porcine kidney-ureter unit. The authors demonstrated face, content, construct validity of both their curricula, as per the old taxonomy of validation studies [15]. Similarly, the European Association of Urology Urolithiasis Section (EULIS) has also developed a URS curriculum and exam for novice residents [16] and report positive outcomes [17].

The models employed in this curriculum were the URO Mentor VR simulator, Uro-Scopic Trainer, Advanced Scope Trainer and FFCs. The content validity results of this study show that, despite some benefits [18, 19], VR is still rated relatively inferior to other modalities. Although rated highly by several validation studies, the URO Mentor [10] was developed over two decades ago and the technology is likely outdated but is also still very costly. Nevertheless, if already available, it may be beneficial in grasping concepts, familiarizing with instruments, cognitive preparation and for its noted additional benefits of fluoroscopy [6].

Despite human cadavers being perceived as the most realistic and gold standard modality of training, their use is limited due to cost, licencing and lack of availability [20]. Participants in this study also rated FFCs very highly but no statistically significant differences were identified between the FFC and non-FFC cohorts in the OR. OSATS scores were noted to be particularly low during FFCs, perhaps due to difficulty in ureteric navigation, as stated by participants. As such, cadavers may not be suitable for training at the novice phase of training and certainly not cost-effective to utilise in an isolated manner for a single procedure. Rather, live animal or cadaveric simulation can take place at the later stages of training in the form of “masterclasses” alongside other procedures to make full use of them, and benefit from anatomical variation [21]. The British Association of Urological Surgeons cadaveric training programme [22] is an example initiative.

The two dry-lab models utilised in our curriculum were also highly rated by participants and seem to sufficient. Synthetic bench models are particularly useful as they provide tangible feedback and enable the use of real instruments and/or irrigation [19]. This study also found that instrument handling was one of the most highly rated aspects of using bench models. These have similar benefits to FFCs but cost significantly less, enable inserting stones for extraction and allow for use of real equipment such as lasers. But, as with FFCs, assessment and feedback are dependent on live or recorded observers.

This study also has several limitations. Although a power calculation was performed for the main RCT (24 required) and recruitment of participants far exceeded this number [9], our results may have been different with a higher number of participants. Furthermore, there was a 30% dropout of follow-up OR data. Of these, the non-VR (*n* = 5) and FFC (*n* = 9) groups were inferior in number compared to the VR and Bench groups. This may have caused bias and skewed our results. Additionally, live OSATS rating scores were a cluster of scores obtained from different faculty members. Moreover, since several parameters could not be assessed through videos, this may have also affected our results. Hence, a mean of all three scores were utilised for the remainder analyses.

Finally, although numerous studies have proved that there is a degree of OR-transferability from simulation training, these have mostly been underpowered small-scale studies conducted on medical students [23]. To our knowledge, SIMULATE is the first RCT to recruit a large number of participants and follow them for a considerable period of time in the OR to test the widely cited hypothesis that simulation can significantly reduce the initial phase of the learning curve [9].

## Conclusions

The SIMULATE URS training curriculum received high educational value from participants, who reported that it significantly improved their skills and provided transferrable skills. Statistically significant improvement was observed with consecutive cases throughout the curriculum and the first OR performance in both semi-rigid and flexible URS. No statistically significant differences were identified with the additional use of fresh frozen cadavers and the URO Mentor VR simulator. A moderate level of correlation was noted on the video OSATS assessments, between two expert assessors, but the poor agreement with a live rating. Participants will be followed up in the OR for transfer validity and compared to an arm with no simulation experience, as part of the SIMULATE randomised controlled trial.

## Supplementary Information

Below is the link to the electronic supplementary material.

Supplementary file1 (PDF 230 KB)

Supplementary file2 (PDF 154 KB)
