# Supplementary material for: The SIMULATE ureteroscopy training curriculum: educational value and transfer of skills
Source: World J Urol. 2021 Feb 3;39(9):3615–21. doi: 10.1007/s00345-021-03604-w (PMC8510983; doi:10.1007/s00345-021-03604-w)

Supplementary Figure: Content validity survey results of all the utilised training modalities (Likert scale 1-5). Abbreviations: UO- ureteric orifice

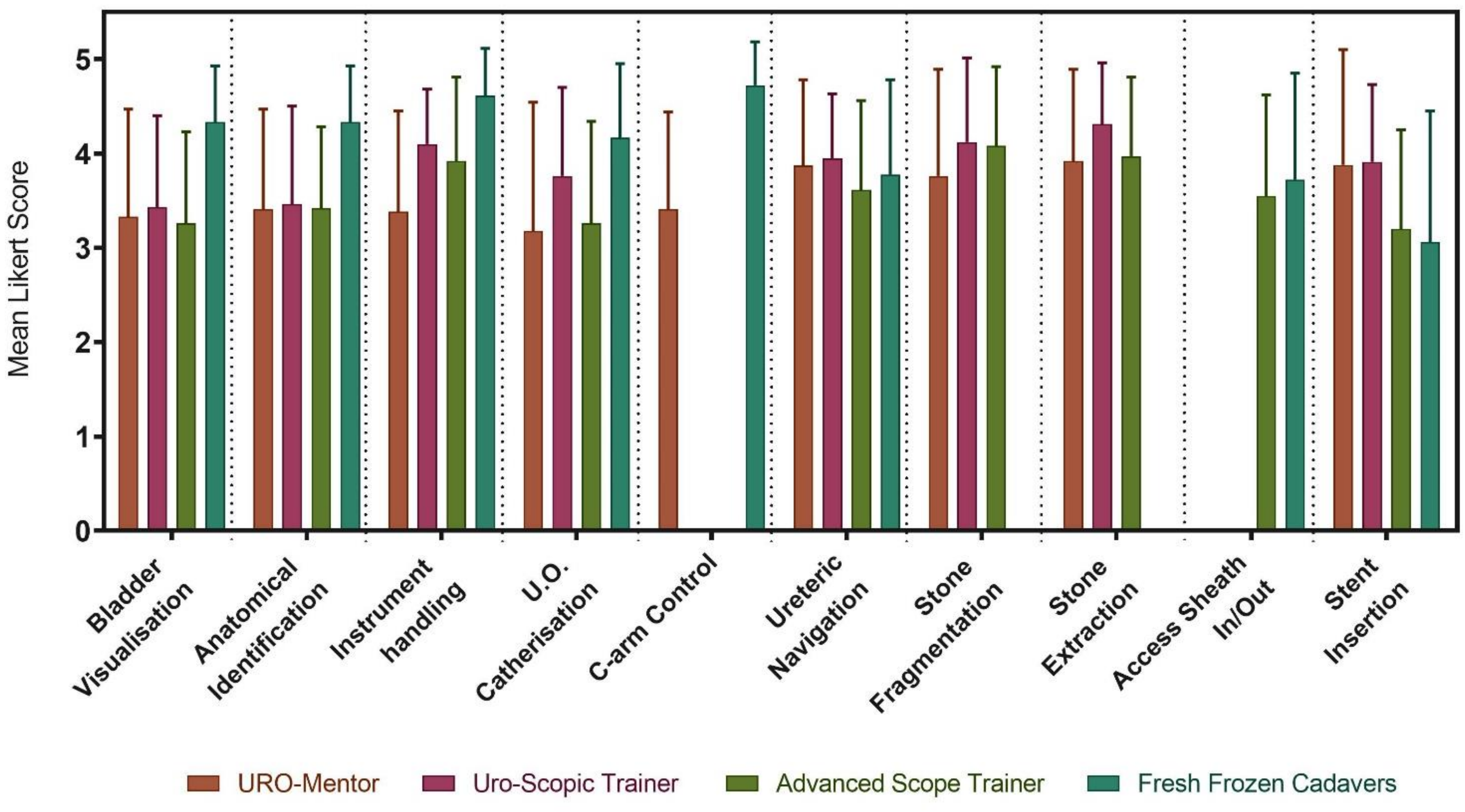

Supplement: Supplementary file 1 — Supplementary file1 (PDF 230 KB) [file 345_2021_3604_MOESM1_ESM.pdf]
