# Supplementary material for: The SIMULATE ureteroscopy training curriculum: educational value and transfer of skills
Source: World J Urol. 2021 Feb 3;39(9):3615–21. doi: 10.1007/s00345-021-03604-w (PMC8510983; doi:10.1007/s00345-021-03604-w)
Supplement: Supplementary file 2 — Supplementary file2 (PDF 154 KB) [file 345_2021_3604_MOESM2_ESM.pdf]

KING'S College LONDON

SIMULATE
Simulation in Urological Training & Education

SIMULATE Evaluation Questionnaire

-Your responses will be processed anonymously-

Name: ……………………………………………………………………………   Age: ………………..

Gender: M/F

Clinical Experience:

Resident/Trainee – stage of training:

Consultant – years of independent practice:

Please indicate the **number of times** you have independently performed the following procedures:

**Procedures**	**No. of times**
**Rigid cystoscopy**	
**Flexible cystoscopy**	
**Semi-rigid Ureteroscopy**	
**Flexible Ureterorenoscopy**	

How useful were the following **lectures**?   (1-not useful, 5-very useful)

1. Initial theatre set up, ureteric access and retrograde studies   1☐ 2☐ 3☐ 4☐ 5☐
2. Guidewires, access sheaths, stents & baskets   1☐ 2☐ 3☐ 4☐ 5☐
3. Lasers – types, size, settings & safety   1☐ 2☐ 3☐ 4☐ 5☐
4. Semi-rigid ureteroscopy   1☐ 2☐ 3☐ 4☐ 5☐
5. Flexible ureterorenoscopy   1☐ 2☐ 3☐ 4☐ 5☐

Comments: ……

………

………

How useful was the **URO-Mentor VR Sim** in teaching the following: (1-not useful, 5-very useful)

1. Bladder Visualisation (Cystoscopy)	1☐	2☐	3☐	4☐	5☐
2. Anatomical Identification	1☐	2☐	3☐	4☐	5☐
3. Instrument handling	1☐	2☐	3☐	4☐	5☐
4. Ureteric Orifice Catheterisation	1☐	2☐	3☐	4☐	5☐
5. C-arm Control	1☐	2☐	3☐	4☐	5☐
6. Ureteric Navigation	1☐	2☐	3☐	4☐	5☐
7. Stone Fragmentation	1☐	2☐	3☐	4☐	5☐
8. Stone Extraction	1☐	2☐	3☐	4☐	5☐
9. Stent Insertion	1☐	2☐	3☐	4☐	5☐

Comments: ……

………

………

How useful was the **Uro-Scopic Trainer** in teaching the following: (1-not useful, 5-very useful)

1. Bladder Visualisation (Cystoscopy)	1☐	2☐	3☐	4☐	5☐
2. Anatomical Identification	1☐	2☐	3☐	4☐	5☐
3. Instrument handling	1☐	2☐	3☐	4☐	5☐
4. Ureteric Orifice Catheterisation	1☐	2☐	3☐	4☐	5☐
5. Ureteric Navigation	1☐	2☐	3☐	4☐	5☐
6. Laser Stone Fragmentation	1☐	2☐	3☐	4☐	5☐
7. Stone Extraction	1☐	2☐	3☐	4☐	5☐
8. Stent Insertion	1☐	2☐	3☐	4☐	5☐

Comments: ……

………

………

How useful was the **Scope/Adv. Scope Trainer** in teaching the following: (1-not useful, 5-very useful)

1. Bladder Visualisation (Cystoscopy)	1☐	2☐	3☐	4☐	5☐
2. Anatomical Identification	1☐	2☐	3☐	4☐	5☐
3. Instrument handling	1☐	2☐	3☐	4☐	5☐
4. Ureteric Orifice Catheterisation	1☐	2☐	3☐	4☐	5☐
5. Insertion/Removal of Access Sheath	1☐	2☐	3☐	4☐	5☐
6. Ureteric Navigation	1☐	2☐	3☐	4☐	5☐
7. Laser Stone Fragmentation	1☐	2☐	3☐	4☐	5☐
8. Stone Extraction	1☐	2☐	3☐	4☐	5☐
9. Stent Insertion	1☐	2☐	3☐	4☐	5☐

Comments: ……

………

………

How useful was the **Cadaveric Simulation** in teaching the following: (1-least useful, 5-very useful)

1. Bladder Visualisation (Cystoscopy)	1☐	2☐	3☐	4☐	5☐
2. Anatomical Identification	1☐	2☐	3☐	4☐	5☐
3. Instrument handling	1☐	2☐	3☐	4☐	5☐
4. Ureteric Orifice Catheterisation	1☐	2☐	3☐	4☐	5☐
5. C-arm Control	1☐	2☐	3☐	4☐	5☐
6. Insertion/Removal of Access Sheath	1☐	2☐	3☐	4☐	5☐
7. Ureteric Navigation	1☐	2☐	3☐	4☐	5☐
8. Stent Insertion	1☐	2☐	3☐	4☐	5☐

Comments: ....

....

....

	(1=strongly disagree, 5=strongly agree)				
This training has significantly improved my skills:	1☐	2☐	3☐	4☐	5☐
I have gained transferrable skills for the operating room:	1☐	2☐	3☐	4☐	5☐
I felt the course was very productive and enjoyable:	1☐	2☐	3☐	4☐	5☐
Simulation-based training and assessment is essential for patient safety:	1☐	2☐	3☐	4☐	5☐
There is a role for further procedural simulation curricula in urology training:	1☐	2☐	3☐	4☐	5☐

Any other Comments:

....

....

....

....

Thank you for your kind participation!
